# Thin films with high surface roughness: thickness and dielectric function analysis using spectroscopic ellipsometry

**DOI:** 10.1186/2193-1801-3-82

**Published:** 2014-02-12

**Authors:** Daniel Lehmann, Falko Seidel, Dietrich RT Zahn

**Affiliations:** Semiconductor Physics, Technische Universität Chemnitz, 09107 Chemnitz, Germany

**Keywords:** Ellipsometry, Roughness, Thickness, AFM, RMS, Dielectric function, PTCDI

## Abstract

An optical surface roughness model is presented, which allows a reliable determination of the dielectric function of thin films with high surface roughnesses of more than 10 nm peak to valley distance by means of spectroscopic ellipsometry. Starting from histogram evaluation of atomic force microscopy (AFM) topography measurements a specific roughness layer (RL) model was developed for an organic thin film grown in vacuum which is well suited as an example. Theoretical description based on counting statistics allows generalizing the RL model developed to be used for all non-conducting materials. Finally, a direct input of root mean square (RMS) values found by AFM measurements into the proposed model is presented, which is important for complex ellipsometric evaluation models where a reduction of the amount of unknown parameters can be crucial. Exemplarily, the evaluation of a N,N’-dimethoxyethyl-3,4,9,10-perylene-tetracarboxylic-diimide (DiMethoxyethyl-PTCDI) film is presented, which exhibits a very high surface roughness, *i.e.* showing no homogeneous film at all.

## Background

Spectroscopic ellipsometry (SE) is a commonly used tool to determine the thickness of thin films and investigate their dielectric function in a non-destructive way. During SE measurements the reflection properties of the whole sample are recorded. A subsequent evaluation by modeling the measured data is necessary to extract properties of individual layers. This procedure requires some knowledge of the general sample structure: the dielectric function of the substrate should be known with high precision and also the order of layers (*e.g.* substrate/oxide/film 1/film 2/roughness) is a necessary input parameter. The more parameter values are unknown in the beginning of the evaluation, the more complex is the evaluation and eventually no solution might be found if some unknown parameters are highly correlated. A possible solution is a step by step analysis of the sample during the preparation, *i.e.* by recording SE spectra after growth of each individual layer. As roughness is a side effect of layer growth itself, this procedure cannot be used for the determination of surface roughness. Another way of reducing the number of unknown parameters is the inclusion of results from other techniques as, for example, UV–vis spectroscopy. Here, we report on the inclusion of AFM topography measurements to support the evaluation of SE spectra of thin film samples with high surface roughness. We then generalize the RL model to be useful for any evaluation of surface roughness of thin films grown on substrates. Finally, it is shown how the root mean square (RMS) determined by AFM measurements can directly be used as input parameter during SE evaluation.

## Methods

**Figure 1:**
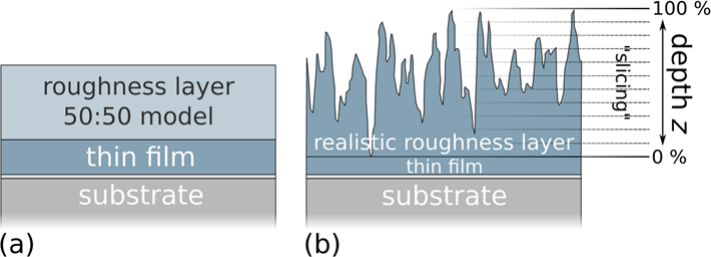
**Roughness layer model comparison. (a)** Common ellipsometry evaluation model: the RL is treated as an effective medium approximation (EMA) of 50% material and 50% void. **(b)** Realistic roughness layer derived from a cross section through an AFM picture of a DiMethoxyethyl-PTCDI film (Figure 2a). By z-slicing the RL into infinitesimal thin slices and treating each slice with an individual EMA material ratio, a more precise RL model can be derived.

The common way to handle a rough film in ellipsometric modeling is to divide it into two parts: one part containing the homogeneous film and the other part representing a non-homogeneous form referred to as "roughness layer" (Figure 1a).

The first part is simulated with the physically meaningful dielectric function belonging to the material. The determination of this dielectric function is usually the aim of the evaluation once the layer thicknesses have been determined. The second part is typically simulated using an effective medium approximation (EMA), a mixture of 50% material – having the dielectric function of the first part of the simulated layer – and of 50% void space with the dielectric function of vacuum (further referred to as "50:50 model") (Gordan et al. 2006a, b; Louis et al. 2007; Volintiru et al. 2008). However, this estimation of the mixture ratio is not precise. The realistic roughness topography of grown layers has a profile comparable to mountains: if one virtually cuts a volume segment into slices of different height, at the height of the valleys the amount of material in each slice is high. At the height of the mountain tips, however, the amount of material is almost zero in each slice (Figure 1b). Hence, the material to void ratio is not constant throughout the roughness part of the layer, but changes as a function of distance to the virtual interface between the RL and the homogeneous part. A similar approach was already discussed by Aspnes et al. ( 1979), but with various geometrically constructed roughness profiles instead of AFM measured profiles.

The consequence of using a simple 50:50 model is an incorrect dielectric function of the roughness part and, hence, an incorrect dielectric function of the homogeneous part as both parts are correlated during the evaluation. These inaccuracies are small for transparent thick films with a small surface roughness. However, if the thickness of the RL has the same order of magnitude as the homogeneous film thickness, a reliable determination of the dielectric function is not possible.

**Figure 2:**
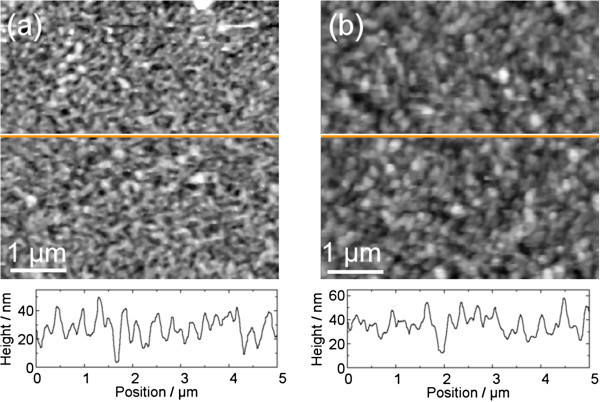
**AFM measured topographies. (a)** Height profile of DiMethoxyethyl-PTCDI: grayscale from 0 nm (black) to 58 nm (white), **(b)** height profile of DiPhenyl-PTCDI for comparison: grayscale from 0 nm (black) to 94 nm (white). The roughness parameter, *i.e.* the material/void function, is deduced by integrating over a grayscale histogram of each AFM picture.

AFM is a method to determine the topography of a sample surface. Commonly, an RMS value is given as value for the surface roughness. For determination details see Méndez-Vilas et al. ( 2002). However, much more information can be deduced for creating a sophisticated roughness model for SE. Exemplarily, an extreme case should be discussed: the dielectric function of an organic thin film of DiMethoxyethyl-PTCDI with a very high surface roughness is determined. A more detailed view on this semiconducting molecule itself and the implication of high surface roughness on electrical behavior when used in organic field-effect transistors can be found elsewhere (Lehmann and Zahn 2009). The thin films were prepared by organic molecular beam deposition under high vacuum conditions (*p*_base_ < 5 × 10^-7^ mbar) on Silicon/SiO_2_ (1.4 nm) substrates. The nominal thickness, respectively the deposited mass, during growth was monitored by a quartz micro balance (QMB). A nominal thickness of 30 nm, based on known QMB ratios for other PTCDI derivatives forming smooth films, was the deposition target. The SE measurement was performed 47 days after the film deposition using a Woollam VASE Ellipsometer, measuring at three incidence angles (65°, 70°, 75°) in a spectral range from 0.74 eV to 4.50 eV. The depolarization was measured additionally using an M2000 T-Solar Ellipsometer from Woollam in the same spectral range. Figure 2a shows an AFM picture of DiMethoxyethyl-PTCDI. As comparison a second picture of another molecule, N,N’-diphenyl-3,4,9,10-perylene-tetracarboxylic-diimide (DiPhenyl-PTCDI, nominally 30 nm thick), is also shown in Figure 2b. The comparison later demonstrates the error tolerance of the method with regard to a wrong input.

## Results and discussion

**Figure 3:**
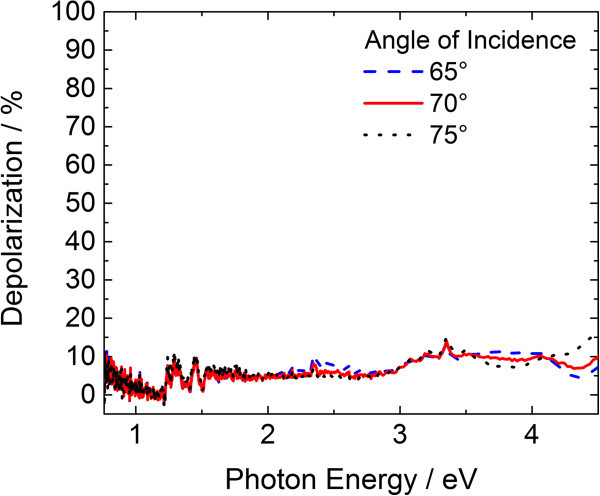
**Depolarization of the DiMethoxyethyl-PTCDI sample during ellipsometry measurement.**

For the method presented, the presumption has to be made that the Fresnel relations are valid. This is true for wavelengths much larger than the RL thickness and structure periodicity. Hence, the light scattering has to be weak and depolarization can be ignored. In the presented case the RL as well as the periodicity are in the range of 100 nm. As the film and RL thicknesses are determined in the transparent region below 1.4 eV (λ ≥ 886 nm) we consider the Fresnel relations valid here. Figure 3 shows that the depolarization is low even for higher energies.

**Figure 4:**
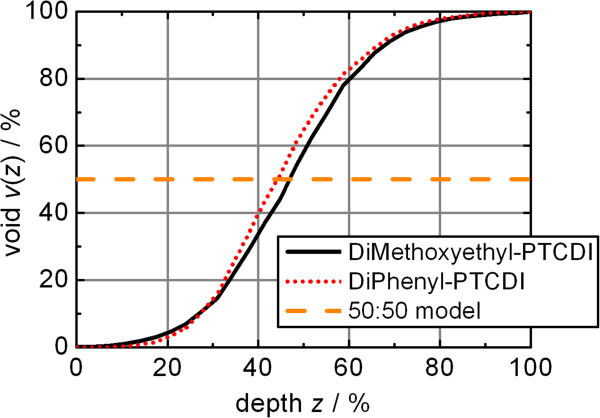
**Material/void ratio comparison.** DiMethoxyethyl-PTCDI and DiPhenyl-PTCDI ratios are deduced from the AFM topography measurements of Figure 1.

Slicing the measured volume segment into equal height slices as described above can be performed by taking a histogram of the grayscale AFM pictures. Each grey level corresponds to a specific height slice. The amount of pixels with the same grey level determines the amount of material in the corresponding height slice. An integration over all grey levels leads to a material/void ratio as a function of the height position within the RL (Figure 4).

The resulting material/void function can be fitted by the Boltzmann equation1

where *v*(*z*) is the percentage of voids as a function of the dimensionless depth *z*, starting from the homogeneous film with *v*(*z* = 0%) = 0%. The depth is also given as percentage as the thickness of the film is variable during fitting while SE modeling is performed. The resulting parameters are compared in Table 1; they are similar for both films.

**Table 1:** **Boltzmann equation parameters determined by fitting the AFM derived material/void ratios**

	***A***/ %	***B***/ %	***C***/ %	***D***/ %
DiMethoxyethyl-PTCDI	-1.5	100.3	46.6	9.7
DiPhenyl-PTCDI	-2.8	99.7	44.0	9.3

The Boltzmann equation with the determined parameters is transferred into an SE RL model of the layer utilizing a Bruggeman EMA (Bruggeman 1935) in the WVASE32 software (J.A. Woollam Co.). The homogeneous layer and RL thicknesses were determined using a Cauchy model in the range of 0.76 eV to 1.40 eV. Table 2 compares the results of the deduced material/void functions (layer order: Si/SiO_2_/homogeneous film/AFM derived RL) to the results of the 50:50 model (layer order: Si/SiO_2_/homogeneous film/50:50 model RL).

**Table 2:** **Thicknesses** ***d*** **of a homogeneous DiMethoxyethyl-PTCDI film below the surface roughness and the corresponding roughness layer heights** ***d***_**r**_ **determined for a film with the use of different roughness profiles**

***Roughness profile of***	***d***/nm	***d***_r_/nm
DiMethoxyethyl-PTCDI (AFM)	0	107.6 ± 0.3
DiPhenyl-PTCDI (AFM)	0	104.2 ± 0.3
Proposed model with *σ* = 13.5%	0	94.8 ± 0.3
50:50 model	27 ± 76	39 ± 70

The results from the material/void functions are very similar and predict both a Volmer-Weber type film growth with no homogeneous film below. The 50:50 model yields only uncertain values where a small change of starting parameters results in significantly different thicknesses. The peak valley distance – identical to the rough layer thickness in the material/void function model – is predicted to be larger than the AFM measured height profile. This can be explained with the limited resolution of the AFM tip: with a diameter of at least 10 nm only peaks and valleys of the same scale and above can be resolved.

Even though the model was derived from AFM pictures the depth *z* was included as a percentage and not as a thickness value in order to be generally applicable. Therefore, the shape of the material/void function does not depend on the actual thickness. This is especially supported by the results of the DiPhenyl-PTCDI topography measurements: although the AFM height profile exhibits a peak-to-valley distance almost twice as large as for the DiMethoxyethyl-PTCDI, the derived material/void function yields very comparable results during thickness determination in SE.

**Figure 5:**
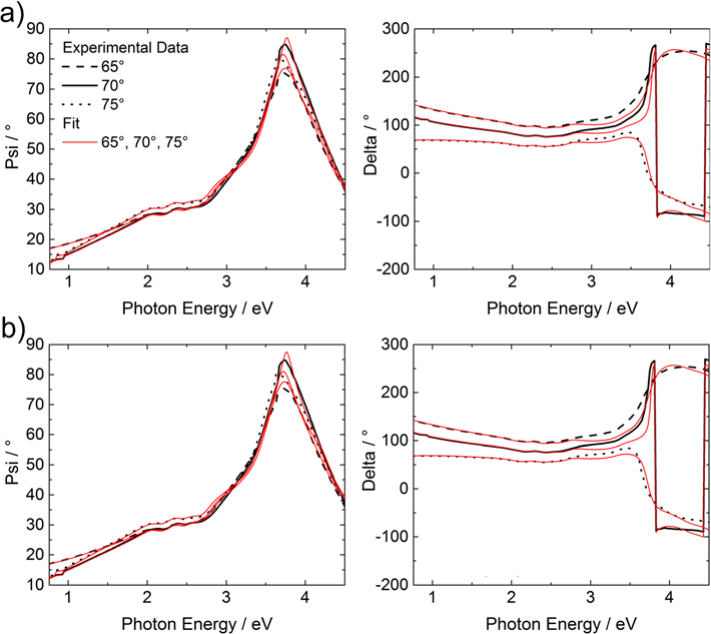
**The ellipsometric angles** ***ψ*** **and** ***Δ*** **and the corresponding fits with general oscillator model. (a)** Fit of the 50:50 model, **(b)** fit of the AFM derived roughness profile model.

**Figure 6:**
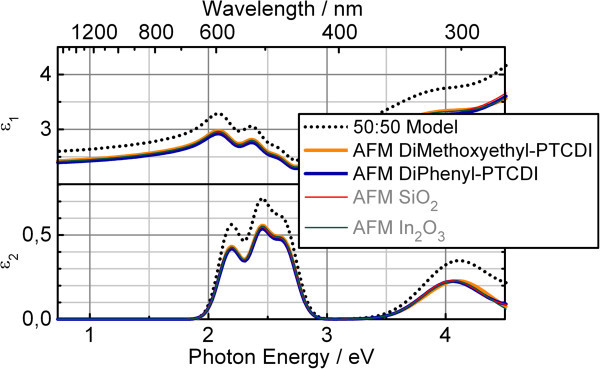
**Dielectric functions determined for a DiMethoxyethyl-PTCDI rough thin film using different roughness profiles.** Dotted curve: dielectric function determined using the conventional 50:50 EMA model. Orange line: dielectric function determined using the actual AFM profile of the film (parameters in Table 1). Other lines: dielectric functions for the same DiMethoxyethyl-PTCDI film, but using the AFM roughness profiles of completely different films. The AFM derived roughness profiles yield almost identical results independent of their manifold origin, whereas the common 50:50 model overestimates the dielectric function.

The determined thicknesses are used as input during the determination of the dielectric function. Therefore, a general oscillator model was utilized. Figure 5 shows the fits to ψ and Δ for the 50:50 model and the presented AFM derived model. Figure 6 compares the results. Even though the quality of the fit does not differ significantly, the 50:50 model overestimates the dielectric function. Additionally, the error bar has to be considered to be as large as for the thickness determination: this result is not reliable. The material/void function model results for the dielectric functions deviate only slightly. The dielectric function of the AFM derived material/void function of DiMethoxyethyl-PTCDI itself is the most reliable one.

Not shown in detail are further stress tests of the material/void function model: AFM pictures of thin films with a large range of thicknesses and from various sources where converted to material/void functions as presented above. Exemplarily, results from SiO_2_ (1.23 nm peak to valley distance)^a^ and In_2_O_3_ (200 nm peak to valley distance) (Dhananjay et al. 2008) are also shown in Figure 6. These intentionally wrong inputs yield dielectric functions of DiMethoxyethyl-PTCDI with even less deviation from the actual DiMethoxyethyl-PTCDI material/void function compared to the DiPhenyl-PTCDI material/void function.

Based on these findings we propose a general RL model to be used instead of the 50:50 model for grown thin films:

The deposition of a large number of particles on a flat surface with random distribution of particles inside the deposition beam will yield a normal distribution, if converted to a histogram and integrated as described above, as long as surface diffusion is neglected:2

The value *λ* is the expected value and *k* is the number of particles sticking to the surface with the probability *N*(*Z* = *k*) at a specific area element having the size of each particle. The expected value equals the variance *E*(*Z*) = *Var*(*Z*) = *λ*. The mean square error (MSE) of this experiment is defined as3

where *m* is the number of particles which can find space on the surface, if only one particle is allowed at each area segment. The corresponding RMS function can be defined as4

Each particle consumes a fraction of area of the surface. Once this area is occupied, the next monolayer starts to grow on top at this specific position. Therefore, *k* is also equal to the number of monolayers, and, hence, *N*(*Z* = *k*) is the probability to find *k* monolayers at a specific position on the substrate. Then, *λ* is the expected number of monolayers. For a discrete distribution as in equation (2) the variance can be calculated as follows:5

Here, *p*_i_ = *m*_i_/*m* is the probability to find a particle at the position *i* on the surface (*m*_i_ being the number of particles at each position *i* and *m* the total number of particles). For a monolayer one particle is considered on each position *i* and, hence, this ratio becomes *p*_i_ = 1/*m*. Then, equation (5) equals equation (3). Therefore, we can write . Since the particle size is small compared to the expected value a continuous distribution can be used instead of equation (2), a Gaussian distribution with *λ* = *C* = *σ*^2^:6

where *z* is the dimensionless height of the film. The resulting material/void function is7

Additionally, in the boundary condition for *z* → ∞ only voids are remaining. This leads to *c* = 1/2. If *C* is defined as 50%, the material/void function equals 50% at a relative depth of *z* = 50%. This is in good agreement with *v*(*z*) found from the evaluation of the AFM measurements. This also ensures that the mean amount of material in the roughness film found by the ellipsometry model equals 50% and, hence, the thickness of the *v*(*z*)-RL remains comparable with thicknesses determined by the common 50:50 model. Additionally, the nominal thickness of the RL can easily be defined as *d*_r,nom_ = *d*_r_/2, which is important for growth rate control using a quartz microbalance.

The factor *σ* determines the slope of *v*(*z*). A value of *σ* = 13.5% matches all the empirically found material/void functions best. The resulting material/void function8

can be included into the ellipsometry software yielding with very little deviation the same dielectric function as the Boltzmann equation discussed above derived from the various materials.

**Figure 7:**
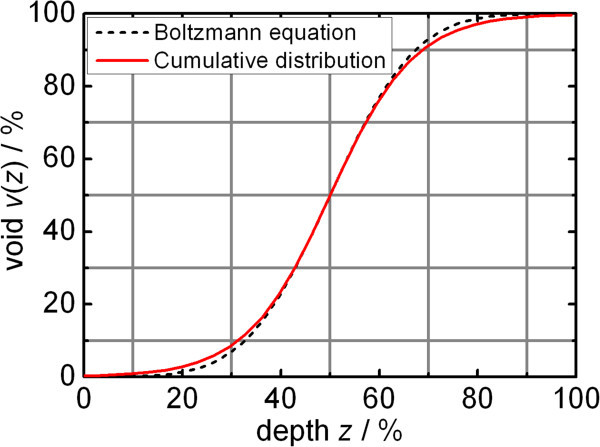
**Comparison between proposed roughness model material/void functions** ***v*** **(** ***z*** **).** The functions formularized with the Boltzmann equation and with the cumulative distribution approach yield comparable results with negligible difference with respect to the evaluation of dielectric functions.

If the ellipsometry software does not allow the use of the error function a similar *v*(*z*) function can also be defined based on the Boltzmann equation (1) with *A* = 0% void at the bottom of the roughness film, *B* = 100% void at the top of the roughness film, *C* = 50% material at the relative depth of *z* = 50%, and *D* = 8.5% as empirically found distribution parameter. The negligible difference to *v*(*z*) based on the cumulative distribution is shown in Figure 7.

Even though the equations introduce multiple new parameters, there is no additional fit parameter during SE evaluation. Only the total thickness of the RL will be fitted as with the 50:50 model before. The values *σ* = 13.5% or *D* = 8.5%, respectively, have also been used very successfully for other material systems, *i.e.* ZnO and Si (will be published elsewhere). There, AFM pictures were unavailable and these values had to be used as starting values. Even without freeing these parameters for fitting, the results were very satisfying. Without having a final proof, these values appear to be reliable starting values in general. Applying the model with these values, the thickness of the DiMethoxyethyl-PTCDI RL is determined to be (94.8 ± 0.3) nm, also without homogeneous film and with the same dielectric function.

RMS values determined by AFM measurements can be transferred into a RL thickness with *v*(*z*) of equation (8) using910

It has to be pointed out that equation (10) only holds for an ideal AFM tip with a diameter of less than the smallest structure of the rough film. In the example of DiMethoxyethyl-PTCDI the thickness of 68.4 nm derived from the RMS value of 9.24 nm deviates from the SE determined film thickness of 94.8 nm by a factor of 1.39. The factor can be seen as a correction factor and might deviate between different AFM tips.

The RL model will not only hold for organic thin films as described exemplarily for DiMethoxyethyl-PTCDI, but as well for all organic and inorganic films grown from a particle beam, where surface diffusion and plasmonic effects can be neglected and the counting statistical approach presented is applicable.

The RL model presented might as well be used for interfaces between two materials, if the underlying material already exhibited surface roughness before. Consequently, *void* has to be substituted by the material grown on top. The formalism remains the same.

Franta and Ohlídal ( 2005) discussed the advantages of Rayleigh-Rice theory (RRT) compared to the EMA used here, especially for laterally large irregularities (*i.e.* islands). Following their discussion, we also recommend to use RRT over EMA for our RL model, if this option is available. However, to our knowledge today’s commercially available software does not offer a sophisticated way for implementing RRT.

## Conclusions

In summary a sophisticated roughness layer model for spectroscopic ellipsometry is developed starting from empirical evaluation of AFM measurements. The model is generalized utilizing statistical considerations to be useful for a broad range of materials exhibiting high surface roughness. The model allows a more precise determination of the dielectric function as well as a more reliable determination of film thicknesses of highly rough materials.

## Endnote

^a^unpublished AFM investigation on SiO_2_ thin films by O. Zywitzki, Fraunhofer FEP Dresden.

## Abbreviations

AFM: Atomic force microscopy
DiMethoxyethyl-PTCDI: N,N’-dimethoxyethyl-3,4,9,10-perylene-tetracarboxylic-diimide
DiPhenyl-PTCDI: N,N’-diphenyl-3,4,9,10-perylene-tetracarboxylic-diimide
EMA: Effective medium approximation
MSE: Mean square error
QMB: Quartz micro balance
RL: Roughness layer
RMS: Root mean square
RRT: Rayleigh-Rice theory
SE: Spectroscopic ellipsometry.
